# Association between the dietary index for gut microbiota and osteoporosis among middle-aged and older adults in the United States

**DOI:** 10.1016/j.pmedr.2025.103212

**Published:** 2025-08-15

**Authors:** Yiming Zhan, Yuhang Liu, Jialing Tang, Siyao Gao

**Affiliations:** a Department of Physical Education, Central South University, Changsha 410083, PR China; b School of Physical Education and Sports, Central China Normal University, Wuhan 430079, PR China

**Keywords:** Dietary index for gut microbiota, DI-GM, Osteoporosis, NHANES, Bone mineral density, Middle-aged and elderly population

## Abstract

**Objective:** Osteoporosis is an age-related disease, and the gut microbiota plays a crucial role in regulating bone mineral density (BMD) through modulating nutrient absorption, immunity, and bone metabolism. This research examines the association between the dietary index for gut microbiota (DI-GM) and osteoporosis prevalence among US middle-aged and older adults.

**Methods:** We included 7255 middle-aged and elderly adults from the National Health and Nutrition Examination Survey (NHANES) 2007–2020. The DI-GM was calculated based on 14 dietary components associated with gut microbiota health. Osteoporosis was defined by femoral neck BMD T-score ≤ −2.5. Multivariable logistic regression and restricted cubic spline (RCS) models were employed to examine the relationship between DI-GM and osteoporosis.

**Results:** Higher DI-GM scores were significantly and negatively associated with the prevalence of osteoporosis (odds ratio [OR] = 0.91, 95 % CI = 0.85–0.97), and a nonlinear trend was observed. Additionally, a higher beneficial component score of DI-GM was associated with a lower incidence of osteoporosis (OR = 0.85, 95 % CI = 0.78, 0.92). Sensitivity analyses further confirmed the robustness of these findings.

**Conclusions:** Higher DI-GM scores were significantly and nonlinearly associated with a lower prevalence of osteoporosis. Future research should validate these findings through longitudinal studies.

## Highlights

• This study first explores diet-related gut microbes and bone health in adults.
• The analysis includes 19,774 adults, representing 42.2 million individuals.
• A nonlinear link exists between gut microbe-related diet and osteoporosis.

## 1 Introduction

Osteoporosis is a skeletal disorder characterized by reduced bone mineral density (BMD) and diminished bone mass, which increases the risk of fracture (National Institutes of Health (NIH), 2001). It is typically diagnosed when the BMD T score ≤ −2.5 (Kanis, 1994). Fractures resulting from osteoporosis become more prevalent in women after age 55 and in men after age of 65, leading to significant musculoskeletal morbidities, increased mortality rates, and higher healthcare costs (Compston et al., 2019). With the aging global population, the prevalence of osteoporosis is increasing worldwide. Recent systematic reviews estimate that osteoporosis affects 18.3 % of individuals globally, with the highest prevalence observed among older adults and postmenopausal women (Salari et al., 2021). Severe fractures and malnutrition are also major causes of death in the elderly (Center et al., 1999; Lancet, 2017; GBD 2016 Mortality Collaborators, 2017). Despite the growing epidemic trend and significant disease burden, there are currently no recommended preventive measures that effectively thwart osteoporosis. Therefore, identifying dietary factors associated with osteoporosis in middle-aged and elderly individuals is essential for its prevention and management.

The dietary index for gut microbiota (DI-GM) is an integrated dietary index proposed by Kase et al., based on a comprehensive review of 106 studies examining the relationship between diet and the gut microbiome. This analysis identified 14 dietary factors that can positively or negatively affect the microbiome. The DI-GM serves as a tool for evaluating dietary quality to promote a healthy gut microbiome (Kase et al., 2024). It also helps individuals understand how dietary patterns influence the microbiome, enabling more informed dietary choices to enhance metabolism, improve gut health, and prevent diet-related diseases (Ross et al., 2024). A growing body of epidemiologic and preclinical evidence supports the important role of gut microbiota and dietary components in the prevention of osteoporosis and its associated mortality (Duffuler et al., 2024; Lindsay, 1994; Claesson et al., 2012). Notably, the individual components of the DI-GM have demonstrated relationships with bone health. For instance, whole grains, identified as beneficial components of the DI-GM, have been associated with a lower risk of low-trauma fractures in population-based cohort studies, particularly among older women (Langsetmo et al., 2011). In contrast, processed meats, deemed detrimental within the DI-GM framework, have been correlated with osteoporosis through a genetic association linking dietary habits involving processed meats to bone health, as evidenced by a Mendelian randomization study (Xu et al., 2022). Furthermore, numerous studies have established a connection between the gut microbiome and bone health, highlighting the microbiome as a promising target for osteoporosis therapies (Mimee et al., 2016). However, while individual dietary components have been widely studied in relation to osteoporosis and BMD, the role of DI-GM, in influencing skeletal health remains underexplored.

Given the close relationship between diet and osteoporosis, this study aimed to investigate the association between DI-GM and the risk of osteoporosis in middle-aged and older adults using data from the National Health and Nutrition Examination Survey (NHANES).

## 2 Methods

### 2.1 Data sources

We used data from seven consecutive NHANES cycles (2007–2020) because these cycles included the key variables required for this study. NHANES represents one of the most comprehensive health-related initiatives undertaken by the National Center for Health Statistics (NCHS) in collaboration with the Centers for Disease Control and Prevention, with data periodically released for public access. The survey comprises cross-sectional assessments designed to yield nationally representative data concerning the civilian, non-institutionalized population of the United States. The data collection protocol received approval from the NCHS Ethics Review Board, and all participants provided informed consent before their interviews and examinations. Consequently, no further institutional review board approval or informed consent was necessary for this investigation.

### 2.2 Study design and population

Our study included a total of 29,659 participants aged 40 years or older, as the risk of osteoporosis increases with age and typically becomes clinically significant after the age of 40 (Ensrud and Crandall, 2017). The exclusion criteria were as follows: individuals missing osteoporosis data (*n* = 13,410) were excluded. To reduce diagnostic heterogeneity and ensure alignment with clinically relevant outcome definitions, Participants with osteopenia were also excluded (Looker et al., 1995). Additionally, those missing any components of the DI-GM (*n* = 504) and individuals with incomplete demographic information, including age, gender, race and ethnicity, marital status, poverty-income ratio (PIR), or educational attainment, were excluded from the analysis. Additional exclusions were applied to participants lacking data on critical health factors, such as body mass index (BMI), alcohol consumption, smoking status, sedentary behavior, sleep duration, total cholesterol, and high-density lipoprotein cholesterol (HDL-C). Consequently, 7255 participants were included in the final analysis (**Supplementary Fig. 1**).

### 2.3 Assessment of dietary index for gut microbiota

According to the scoring criteria established by Kase et al., 14 food items or nutrients were identified as components of the DI-GM. The beneficial components include avocado, broccoli, chickpeas, coffee, cranberries, fermented dairy, fiber, green tea, soybeans, and whole grains (Since NHANES does not record specific types of tea consumption, no data is available). In contrast, red meat, processed meat, refined grains, and high-fat diets (defined as those in which fat constitutes ≥40 % of total energy intake) are considered unfavorable components (Kase et al., 2024). Dietary recall data from the NHANES, covering the years 2007 to 2020, were utilized to compute the DI-GM, with the results presented in **Supplementary Table 1**. For gut-beneficial foods, a score of 1 was assigned if consumption met or exceeded the gender-specific median; otherwise, a score of 0 was recorded. Conversely, for gut-unfavorable foods, a score of 1 was assigned unless consumption fell below the median or 40 % (in the case of high-fat diets), in which case a score of 0 was allocated. The total DI-GM score, which ranges from 0 to 13, integrates both beneficial (0–9) and unfavorable (0–4) effects on gut microbiota. Based on previous literature, DI-GM scores were subsequently categorized into continuous variables and categorical variables (quartiles and divided into beneficial and unfavorable) (Zhang et al., 2024a).

### 2.4 Assessment of osteoporosis

Based on previous research, the NHANES team conducted an independent analysis of BMD levels across various regions of the lumbar spine and femur, including the total femur, femoral neck, trochanter, and intertrochanteric region. BMD was evaluated using a Holopic QDR 4500 A fan-shaped beam densitometer dual-energy X-ray absorptiometry, with measurements quantified in grams per cubic centimeter (g/cm^2^) (Fan et al., 2014). Given that femoral neck BMD is a strong predictor of osteoporotic fractures, all eligible individuals were assessed for osteoporosis or bone loss based on their femoral neck BMD (Kanis et al., 2008). The average bone density of white males and females aged 20 to 29 served as the reference value, incorporating specific values from prior studies (Looker et al., 2012; Looker et al., 1997). Participants with a BMD T-score of less than or equal to −2.5 are classified as having osteoporosis, which corresponds to a BMD of 0.58 g/cm^2^ or lower for men and 0.56 g/cm^2^ or lower for women. Conversely, participants with a BMD T-score of −1 or higher are classified as having normal BMD, specifically men with a BMD of 0.79 g/cm^2^ or higher, and women with a BMD of 0.74 g/cm^2^ or higher. All other cases are categorized as osteoenia. For further details regarding BMD testing, please refer to the NHANES website (Centers for Disease Control and Prevention (CDC), 2021). **Supplementary Table 2** delineates the diagnostic criteria for osteoporosis.

### 2.5 Covariates

Several potential confounding variables were meticulously evaluated, drawing on both existing literature and clinical expertise. These variables encompassed age, gender, race and ethnicity, marital status, educational level, PIR, alcohol consumption, smoking status, BMI, Sedentary behavior, sleep duration, total cholesterol, and HDL-C (Tang et al., 2024; Hou et al., 2023). In the logistic regression analysis, age was treated as a continuous variable. The race categories included non-Hispanic Black, non-Hispanic White, Mexican American, and other races. Marital status was classified into married or living with a partner, never married, and widowed/divorced/separated. Education levels were categorized as high school, some college or higher, or below high school. The PIR was divided into three groups: ≤1.30, 1.30–3.50, and > 3.50. Smoking status was defined using two questions: “Have you smoked at least 100 cigarettes in your lifetime?” and “Do you now smoke cigarettes? (every day, some days, or not at all)” Based on these responses, smoking status was classified as former, never, or current smoker. Alcohol consumption was self-reported and classified into the following categories: never (consumed fewer than 12 drinks in a lifetime), former (consumed at least 12 drinks in a year but not within the past year, or refrained from drinking in the last year but had consumed at least 12 drinks over a lifetime), mild (no more than one drink per day for females and no more than three drinks per day for males), and heavy (at least three drinks per day for males and at least four drinks per day for females). This categorization reflects evidence that different levels of alcohol intake may have different biological effects. Light drinking was associated with increased bone density and a more favorable gut microbiota composition, whereas heavy drinking was associated with bone loss and gut microbial dysbiosis. Former drinkers were retained as a separate group to control for potential residual effects of previous drinking (Feskanich et al., 1999; Qiao et al., 2024). Both HDL-C and total cholesterol levels were considered, as they may have implications for osteoporosis risk (Wang et al., 2024a).

### 2.6 Statistical analysis

All statistical analyses were weighted according to the NHANES sampling design. Continuous variables were presented as means with standard errors (SE), depending on the distribution, and were compared using the *t*-test. Categorical variables were expressed as numerical counts and percentage frequencies (%) and analyzed using weighted Chi-square tests. To examine the relationship between DI-GM and osteoporosis, weighted multivariable logistic regression analyses were conducted. The odds ratio (OR) and its corresponding 95 % confidence interval (CI) were calculated. Two models were fitted: Model 1 was unadjusted model, with no adjustments for covariates. Model 2 was adjusted for age, gender, race/ethnicity, marital status, education level, PIR, BMI, alcohol consumption, smoking status, sedentary behavior, sleep duration, total cholesterol, and HDL-C. Trend tests aimed to explore the linear association between DI-GM group and the risk of osteoporosis. Restricted cubic spline (RCS) was used in weighted logistic regression models to assess the dose-response relationship between DI-GM and osteoporosis. To assess the robustness of the results, a sensitivity analysis was conducted by incorporating additional covariates including diabetes mellitus, hypertension, and cardiovascular disease. All statistical analyses were performed using R software (version 4.4.1) from the R Project for Statistical Computing. Statistical tests were two-sided, with a significance level set at *P* < 0.05.

## 3 Results

### 3.1 Characteristics of the study participants

A total of 7255 participants met the study's inclusion and exclusion criteria, with a mean age of 56.27 ± 0.22 years. Of these, 55.39 % were male and 44.61 % were female. The racial/ethnic composition was as follows: 71.88 % non-Hispanic White, 11.37 % non-Hispanic Black, 10.38 % from other racial groups, and 6.37 % Mexican American. Finally, there are 581(7.49 %) participants with osteoporosis (Table 1). Individuals with osteoporosis were more likely to be older, female, non-Hispanic White, to have lower incomes, higher HDL-C, lower DI-GM and beneficial scores, and higher unfavorable scores.

**Table 1:** Characteristics of adults aged 40 and above with osteoporosis from NHANES 2007–2020 (n = 7255).

Characteristics	Total n(%)/mean(SD)	Without osteoporosis n(%)/mean(SD)	Osteoporosis n(%)/mean(SD)	*P*-value
Age, years	56.27 ± 0.22	55.47 ± 0.21	66.07 ± 0.55	< 0.01
BMI	29.84 ± 0.11	30.16 ± 0.12	25.90 ± 0.27	< 0.01
HDL-C (mg/dL)	52.63 ± 0.31	52.23 ± 0.33	57.61 ± 0.79	< 0.01
Total Cholesterol (mg/dL)	199.41 ± 0.83	199.27 ± 0.89	201.08 ± 2.12	0.44
DI-GM (0–13 items)	4.83 ± 0.03	4.84 ± 0.03	4.72 ± 0.08	0.08
Beneficial to gut microbiota (0–9 items)	2.27 ± 0.03	2.30 ± 0.03	1.96 ± 0.08	< 0.01
Unfavorable to gut microbiota (0–4 items)	2.56 ± 0.02	2.54 ± 0.02	2.76 ± 0.06	< 0.01
Sedentary behavior (min/day)	365.14 ± 4.06	364.30 ± 4.32	375.49 ± 9.02	0.26
Sleep duration (hours/day)	7.07 ± 0.03	7.05 ± 0.03	7.38 ± 0.09	< 0.01
Race/ethnicity				< 0.01
Non-Hispanic Black	1719 (11.37)	1676 (11.97)	43 (3.99)	
Non-Hispanic White	3225 (71.88)	2882 (71.53)	343 (76.18)	
Mexican American	1005 (6.37)	943 (6.40)	62 (5.94)	
Other races	1306 (10.38)	1173 (10.10)	133 (13.89)	
Gender				< 0.01
Female	3108 (44.61)	2689 (42.20)	419 (74.41)	
Male	4147 (55.39)	3985 (57.80)	162 (25.59)	
Education level				< 0.01
Less than high school	1669 (14.28)	1517 (13.80)	152 (20.25)	
High school	1705 (24.73)	1552 (24.43)	153 (28.50)	
College or above	3881 (60.98)	3605 (61.77)	276 (51.25)	
Marital level				< 0.01
Married/Living with partner	4812 (72.17)	4506 (73.37)	306 (57.28)	
Widowed/Divorced/Separated	1882 (21.69)	1646 (20.33)	236 (38.47)	
Never married	561 (6.14)	522 (6.30)	39 (4.25)	
PIR				< 0.01
< 1.3	1831 (15.82)	1660 (15.27)	171 (22.66)	
> 3.5	2731 (53.18)	2582 (54.77)	149 (33.61)	
1.3–3.5	2693 (30.99)	2432 (29.96)	261 (43.73)	
Smoking status				< 0.01
Former	2208 (30.28)	2074 (30.94)	134 (22.16)	
Never	3721 (53.50)	3381 (53.17)	340 (57.55)	
Now	1326 (16.22)	1219 (15.89)	107 (20.29)	
Alcohol consumption				< 0.01
Former	1098 (12.41)	999 (12.07)	99 (16.65)	
Heavy	1204 (16.68)	1154 (17.23)	50 (9.95)	
Never	809 (8.64)	660 (7.73)	149 (19.85)	
Moderate	1118 (18.33)	1050 (18.64)	68 (14.58)	
Mild	3026 (43.93)	2811 (44.33)	215 (38.97)	
DI-GM group				0.27
0–3	1627 (20.49)	1504 (20.41)	123 (21.53)	
4	1697 (22.49)	1550 (22.28)	147 (25.10)	
5	1706 (24.03)	1561 (23.91)	145 (25.50)	
≥ 6	2225 (32.99)	2059 (33.40)	166 (27.87)	
Cardiovascular disease				0.01
No	6286 (88.53)	5812 (88.90)	474 (84.02)	
Yes	969 (11.47)	862 (11.10)	107 (15.98)	
Diabetes mellitus				0.24
Yes	1840 (20.39)	1718 (20.49)	122 (19.18)	
Impaired Fasting Glucose	451 (6.01)	420 (5.94)	31 (6.89)	
Impaired Glucose Tolerance	276 (3.41)	242 (3.23)	34 (5.62)	
No	4688 (70.19)	4294 (70.34)	394 (68.31)	
Hypertension				< 0.05
No	3235 (48.53)	2996 (49.10)	239 (41.60)	
Yes	4020 (51.47)	3678 (50.90)	342 (58.40)	

Footnotes: Continuous variables are presented as mean ± standard error, and categorical variables are presented as n (weighted%). *P*-values were assessed by T-test (continuous variables) or by Chi-square test (categorical variables).The DI-GM score is based on 14 dietary components. The beneficial subscore includes 9 favorable items (range: 0–9), and the unfavorable subscore includes 4 adverse items (range: 0–4). In the DI-GM group, scores of 0–3 indicate the least favorable dietary pattern for gut microbiota health, scores of ≥6 indicate the most favorable dietary pattern, and scores of 4 or 5 reflect intermediate dietary patterns. Sedentary behavior was measured by the self-reported number of minutes spent on sedentary activities (e.g., sitting, lying down) during a typical day.

Abbreviations: BMI, Body mass index; HDL-C, High-density lipoprotein cholesterol; DI-GM, Dietary index for gut microbiota; NHANES, National Health and Nutrition Examination Survey; PIR, Poverty income ratio; SD, Standard deviation.

### 3.2 Association between DI-GM and osteoporosis

**Fig. 1:**
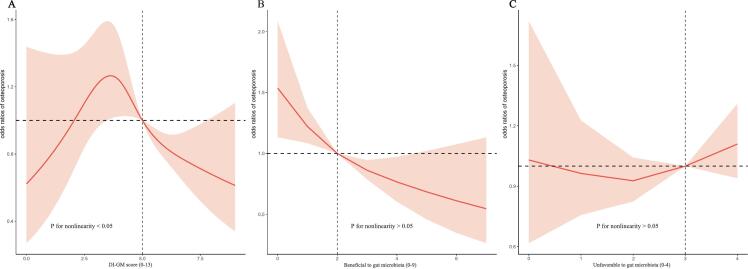
Dose-response relationships between (A) dietary index for gut microbiota (DI-GM), (B) beneficial to gut microbiota, (C) unfavorable to gut microbiota, and the risk of osteoporosis, data from NHANES 2007–2020 (*n* = 7255). The model adjusted for age, gender, race/ethnicity, education level, marital status, PIR, smoking status, sleep duration, HDL-C, total cholesterol, sedentary behavior, BMI and alcohol consumption. Panel A presents the DI-GM as a continuous score ranging from 0 to 13. Panels B and C show subscores based on the number of beneficial (0–9) and unfavorable (0–4) dietary components, respectively. Abbreviations:CI, Confidence interval; NHANES, National Health and Nutrition Examination Survey; PIR, Poverty income ratio; BMI, Body mass index; HDL-C, High-density lipoprotein cholesterol; DI-GM, Dietary index for gut microbiota.

As shown in Table 2, the association between DI-GM and osteoporosis was significant in the fully adjusted model (OR = 0.91, 95 % CI = 0.85, 0.97). In the unadjusted analysis, there was no statistically significant difference in the prevalence of osteoporosis across DI-GM groups (*P* = 0.27). However, after adjusting for DI-GM group, a significant negative correlation was observed between DI-GM values ≥6 and the prevalence of osteoporosis (OR = 0.60, 95 % CI = 0.44, 0.83). Furthermore, the beneficial components of DI-GM significantly reduced the risk of osteoporosis in fully adjusted models (OR = 0.85, 95 % CI = 0.78, 0.92). Additionally, the trend analysis revealed that as DI-GM values increased, the risk of osteoporosis decreased significantly (*P* for trend <0.05). RCS analysis indicated a non-linear relationship between DI-GM and osteoporosis (*P* for non-linearity <0.05), while both beneficial (*P* for non-linearity >0.05) and detrimental (*P* for non-linearity >0.05) components of DI-GM exhibited linear associations with osteoporosis (Fig. 1).

**Table 2:** Association between dietary index for gut microbiota and osteoporosis in adults aged 40 and above from NHANES 2007–2020.

	Unadjusted Model		Adjusted Model	
	OR (95 %CI)	*P*-value	OR (95 %CI)	*P*-value
DI-GM	0.95 (0.90,1.01)	0.09	0.91 (0.85, 0.97)	< 0.05
DI-GM group				
0–3	Reference		Reference	
4	1.07 (0.75,1.52)	0.71	0.89 (0.59, 1.34)	0.58
5	1.01 (0.70,1.47)	0.95	0.93 (0.59, 1.47)	0.76
≥ 6	0.79 (0.60,1.03)	0.09	0.60 (0.44, 0.83)	< 0.05
*P* for trend		< 0.05		< 0.05
Unfavorable to gut microbiota	1.21 (1.09,1.35)	< 0.01	1.05 (0.92, 1.20)	0.47
Beneficial to gut microbiota	0.82 (0.76,0.90)	< 0.01	0.85 (0.78, 0.92)	< 0.01

Footnotes: Adjusted model was adjusted for age, gender, race/ethnicity, education level, marital status, PIR, BMI, smoking status, sleep duration, HDL-C, total cholesterol, sedentary behavior, and alcohol consumption. In the DI-GM group, scores of 0–3 indicate the least favorable dietary pattern for gut microbiota health, scores of ≥6 indicate the most favorable dietary pattern, and scores of 4 or 5 reflect intermediate dietary patterns.

Abbreviations: CI, Confidence interval; DI-GM, Dietary index for gut microbiota; BMI, Body mass index; NHANES, National Health and Nutrition Examination Survey; PIR, Poverty income ratio; HDL-C, High-density lipoprotein cholesterol.

### 3.3 Sensitivity analysis

The results of the sensitivity analysis were consistent with the primary findings. The association between DI-GM and osteoporosis remained significant (All *P*-value <0.05), even after further adjustments for diabetes mellitus, hypertension, and cardiovascular disease, respectively (**Supplementary Table 3**).

## 4 Discussion

In this large cross-sectional study of U.S. adults aged ≥40 years, we found a significant nonlinear inverse relationship between DI-GM score and prevalence of osteoporosis. These findings suggest that greater adherence to a gut microbiota-friendly dietary pattern may be associated with a lower risk of osteoporosis in diverse populations.

Notably, participants with a DI-GM score of 6 or higher exhibited a substantially lower incidence of osteoporosis. This finding may be attributed to the cumulative effect of foods that benefit the microbiome, such as whole grains, soy products, fermented dairy products, and fiber-rich vegetables, all of which are emphasized within the DI-GM framework. These foods have been shown to support gut microbial diversity, facilitate calcium and magnesium absorption, increase the production of short-chain fatty acids (SCFA), and reduce systemic inflammation—mechanisms that are critical for the maintenance of bone health. Epidemiological studies further corroborate this relationship (Rizzoli and Biver, 2018; Claesson et al., 2012; Langsetmo et al., 2012; De Filippis et al., 2016); for example, Langsetmo et al. observed that whole grain intake was associated with a reduced risk of fracture in postmenopausal women (Langsetmo et al., 2012), and Claesson et al. found that fiber-rich diets in older adults promoted a healthy gut microbiota and improved metabolic outcomes (Claesson et al., 2012). Mechanistically, SCFA, particularly butyrate and propionate, produced by gut microbes, regulatory role in bone remodeling by modulating osteoclast and osteoblast activity through immune signaling pathways (Lucas et al., 2018; Tyagi et al., 2018). Thus, high DI-GM scores likely reflect increased SCFA production and enhanced gut-bone axis activity. Conversely, consumption of processed meats, refined grains, and high-fat diets, considered detrimental in the DI-GM, has been associated with dysbiosis and greater bone loss (Perna et al., 2017; De Filippis et al., 2016).

Our findings align with recent laboratory studies demonstrating that prolonged exposure to a high-fat/high-sugar diet induces metabolic alterations across various physiological systems in male mice, including insulin resistance, fasting hyperglycemia, ectopic lipid deposition, and bone degeneration (Burchfield et al., 2018). Furthermore, yogurt is rich in bifidobacteria, and another study has indicated that treatment with bifidobacteria significantly enhances BMD, increases the bone volume/total volume ratio, and elevates trabecular bone count, while effectively mitigating bone loss (Zhang et al., 2024b). Numerous studies have highlighted the differences in gut microbiota diversity between patients with osteoporosis and healthy control groups, particularly regarding dietary improvements (Lin et al., 2023). For instance, the consumption of propolis nanoemulsions has been shown to help maintain the structural and metabolic equilibrium of the gut microbiota, thereby enhancing the efficacy of osteoporosis treatment (Zheng et al., 2024). Furthermore, a cross-sectional study indicated that the Geriatric Nutritional Risk Index serves as an independent risk factor for osteoporosis in the elderly, exhibiting a nonlinear negative correlation with osteoporosis risk in this demographic (Huang et al., 2022). Another cross-sectional study corroborated these findings (Wang et al., 2024b).

From a public health perspective, the DI-GM provides a practical indicator for assessing and promoting dietary habits that benefit gut and bone health. Given the prevalence of suboptimal dietary patterns among middle-aged and older adults, our findings emphasize the potential role of the DI-GM in guiding nutrition-based strategies to reduce osteoporosis risk. Policymakers and health professionals may consider incorporating pro-microbial dietary recommendations into routine prevention strategies, especially for the aging population. This study has several limitations. First, the cross-sectional design precluded causal inference. Second, there may be potential residual confounders due to unmeasured factors or measurement error in the dietary recall data. Third, the DI-GM was calculated from 24-h dietary recalls and may not accurately reflect habitual intake. Future longitudinal studies and randomized controlled trials are necessary to confirm these findings and explore the mechanisms behind them.

## 5 Conclusion

In a nationally representative sample of middle-aged and elderly adults, this study demonstrated a nonlinear inverse association between DI-GM and the prevalence of osteoporosis. These findings suggest that the DI-GM may serve as a practical tool to guide dietary strategies aimed at modulating gut microbiota for the prevention and management of osteoporosis in aging populations. Further research is essential to establish causality and to gain a deeper understanding of the relationship between gut microbiota and bone health.

## CRediT authorship contribution statement

**Yiming Zhan:** Writing – original draft, Methodology, Formal analysis, Data curation. **Yuhang Liu:** Writing – review & editing, Investigation, Conceptualization. **Jialing Tang:** Supervision, Project administration. **Siyao Gao:** Validation, Supervision, Funding acquisition.

## Consent for publication

Not applicable.

## Ethics statement

The research ethics review boards of the National Center for Health Statistics authorized the NHANES study protocols, and all participants provided written informed consent.

## Funding sources

M.Ed. Tang is supported by grant 22YJC890029, the MOE (Ministry of Education in China) Project of Humanities and Social Sciences. The funding bodies had no roles in the design of the study and collection, analysis, and interpretation of data, and in writing the manuscript.

## Declaration of competing interest

The authors declare that they have no known competing financial interests or personal relationships that could have appeared to influence the work reported in this paper.

## Data Availability

The datasets generated and analyzed during the present study are available from the NHANES databases (Available from https://www.cdc.gov/nchs/nhanes/participant.htm).

## Appendix A Supplementary data

Supplementary material **Supplementary Fig. 1.** Flowchart of participant selection, NHANES 2007–2020 for U.S. adults. **Supplementary Table 1.** Components and scoring criteria of the dietary index for gut microbiota in NHANES. **Supplementary Table 2.** Diagnostic criteria for osteoporosis. **Supplementary Table 3.** Sensitivity analysis of the association of the dietary index for gut microbiota with osteoporosis, NHANES 2007–2020 (n = 7,255). Supplementary material
