# Supplementary material for: Association between the dietary index for gut microbiota and osteoporosis among middle-aged and older adults in the United States
Source: Prev Med Rep. 2025 Aug 15;58:103212. doi: 10.1016/j.pmedr.2025.103212 (PMC12398234; doi:10.1016/j.pmedr.2025.103212)
Supplement: Supplementary material — Supplementary Fig. 1. Flowchart of participant selection, NHANES 2007–2020 for U.S. adults. Supplementary Table 1. Components and scoring criteria of the dietary index for gut microbiota in NHANES. Supplementary Table 2. Diagnostic criteria for osteoporosis. Supplementary Table 3. Sensitivity analysis of the association of the dietary index for gut microbiota with osteoporosis, NHANES 2007–2020 (n = 7,255). [file mmc1.docx]

**Supplementary Materials**

**Association between the Dietary Index for Gut Microbiota and Osteoporosis among Middle-Aged and Older Adults in the United States**

**Table of contents**

**[Supplementary Figure 1. Flowchart of participant selection 3](#_Toc11842)**

**[Supplementary Table 1. Components and scoring criteria of the Dietary Index for Gut Microbiota in NHANES. 4](#_Toc27444)**

**[Supplementary Table 2. Diagnostic criteria for osteoporosis.. 5](#_Toc6650)**

**[Supplementary Table 3 Sensitivity analysis of the association of DI-GM with osteoporosis, NHANES 2007-2020 (n = 7,255) 15](#_Toc22656)**


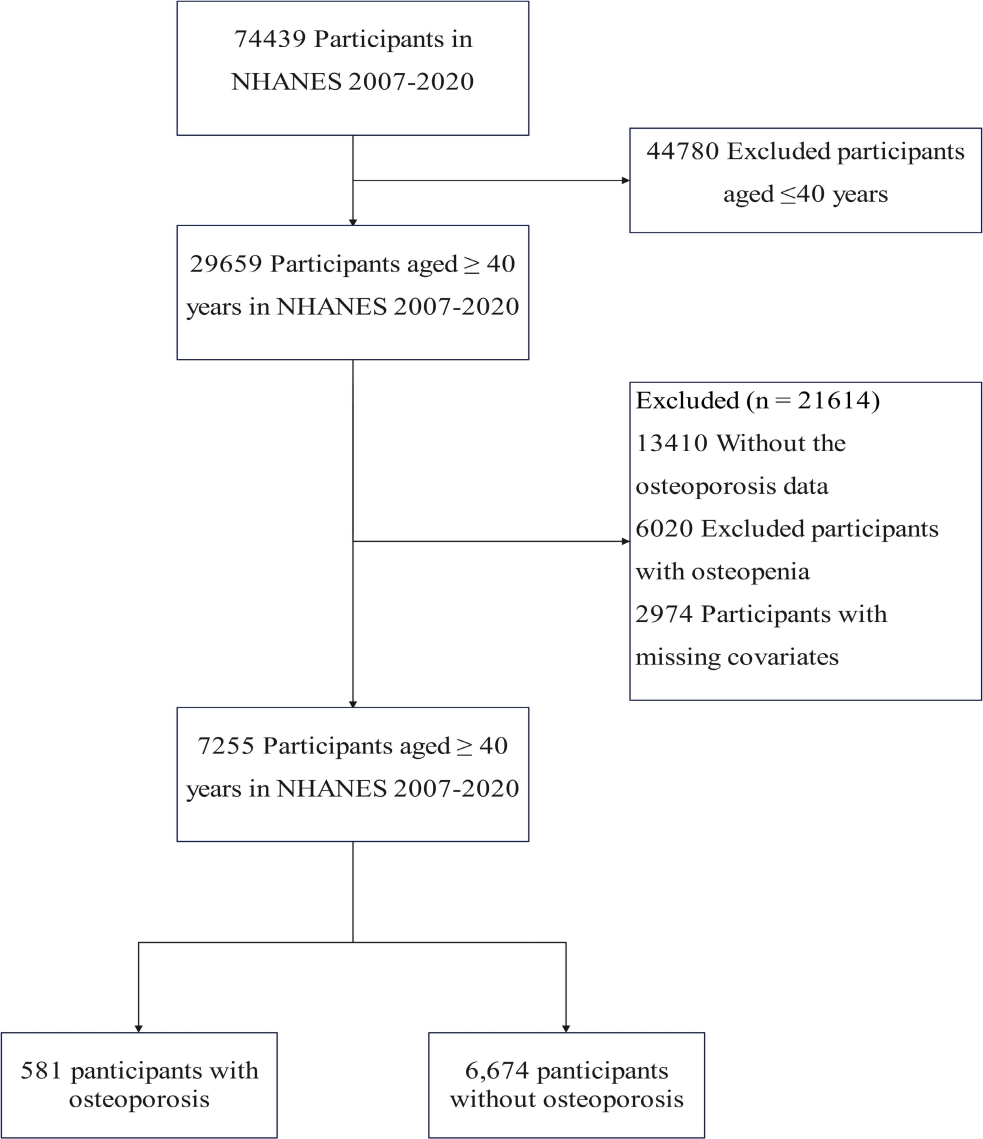


**Supplementary Figure 1. Flowchart of participant selection, NHANES 2007-2020 for U.S. adults.**

Abbreviations: NHANES, National Health and Nutrition Examination Survey

## Supplementary Table 1. Components and scoring criteria of the Dietary Index for Gut Microbiota in NHANES.

| Components of DI-GM | Food items included in NHANES | Scoring criteria |
| --- | --- | --- |
| Beneficial to gut microbiota | Avocados | Score 1 - Consumption≥sex-specific median  Score 0 - Otherwise |
|  | Broccoli |  |
|  | Chickpeas |  |
|  | Coffee |  |
|  | Cranberries |  |
|  | Fermented dairy (including yogurt, cheese, kefir, sour cream, buttermilk) |  |
|  | Fiber |  |
|  | Soybean (including Soy milk, Tofu) |  |
|  | Whole grains |  |
|  | Green tea |  |
| Unfavorable to gut microbiota | Refined grains | Score 0 - Consumption≥sex-specific median  Score 1 - Otherwise |
|  | Processed meat |  |
|  | Red meat |  |
|  | High-fat diet (% energy) | Score 0 - Consumption≥40%  Score 1 - Otherwise |

Abbreviations: DI-GM, dietary index for gut microbiota; NHANES, National Health and Nutrition Examination Survey.

Reference:

1. Zhang X, Yang Q, Huang J, Lin H, Luo N, Tang H. Association of the newly proposed dietary index for gut microbiota and depression: the mediation effect of phenotypic age and body mass index. Eur Arch Psychiatry Clin Neurosci. Published online October 8, 2024. doi:10.1007/s00406-024-01912-x

## Supplementary Table 2. Diagnostic criteria for osteoporosis.

Osteoporosis is a disease characterized by weakened bones and reduced bone density, making fractures more likely. Diagnosis typically involves assessing bone mineral density (BMD) using methods such as Dual-energy X-ray Absorptiometry (DXA), which measures BMD in areas like the spine, hip, and wrist. According to WHO standards, BMD is expressed as a T-score, comparing an individual's BMD to that of healthy young adults. A T-score of ≥ -1.0 indicates normal bone density, -2.5 < T-score < -1.0 suggests low bone mass (osteopenia), and T-score ≤ -2.5 diagnoses osteoporosis. In cases of fragility fractures, osteoporosis may be diagnosed even if BMD results do not meet these criteria. Beyond BMD, physicians evaluate risk factors like age, sex, family history, lifestyle (smoking, alcohol, inactivity), nutrition (calcium and vitamin D intake), and conditions or medications affecting bone metabolism.

1. Looker, A.C., et al., Prevalence of low femoral bone density in older U.S. adults from NHANES III. J Bone Miner Res, 1997. 12(11): p. 1761-8.

**Supplementary Table 3 Sensitivity analysis of the association of dietary index for gut microbiota with osteoporosis, NHANES 2007-2020 (n = 7,255)**

|  | Unadjusted model | | Adjusted Model 1 | | Adjusted Model 2 | | Adjusted Model 3 | |
| --- | --- | --- | --- | --- | --- | --- | --- | --- |
| Variable | OR (95%CI) | *P*-value | OR (95%CI) | *P*-value | OR (95%CI) | *P*-value | OR (95%CI) | *P*-value |
| DI-GM | 0.95 (0.90,1.00) | 0.08 | 0.91 (0.85, 0.96) | < 0.05 | 0.91 (0.85, 0.97) | < 0.05 | 0.91 (0.85, 0.97) | < 0.05 |

Footnotes: The multivariable logistic regression model was adjusted for age, gender, race/ethnicity, education level, marital status, PIR, BMI, HDL-C, total cholesterol, smoking and alcohol consumption. Diabetes mellitus (yes or no), hypertension (yes or no), and Cardiovascular disease (yes or no), were added into the multivariable logistic regression model for additional adjustment, respectively. DI-GM inluded beneficial to gut bicrobiota (0-9) and unfavorable to gut microbiota (0-4).

Abbreviations: CI, Confidence interval; NHANES, National Health and Nutrition Examination Survey; PIR, Poverty income ratio; HDL-C, High-density lipoprotein Cholesterol.
